# Semen quality and bacteriological profile of semen collected by three methods and fertility in Huacaya alpacas (*Vicugna pacos*) inseminated with fresh postcopulatory semen

**DOI:** 10.3389/fvets.2026.1872535

**Published:** 2026-07-10

**Authors:** Nilton Marcial Cardenas Suarez, Wilber García Vera, Danilo Pezo Carreon, Virgilio Machaca Machaca, Valeriano Paucara Ocsa

**Affiliations:** 1 Facultad de Medicina Veterinaria y Zootecnia, Universidad Nacional Micaela Bastidas de Apurimac, Apurimac, Peru; 2 Facultad de Medicina Veterinaria, Instituto Veterinario de Investigaciones Tropicales y de Altura, Universidad Nacional Mayor de San Marcos, Marangani, Peru

**Keywords:** alpaca, artificial insemination, bacteriological profile of alpaca semen, semen collection, semen quality

## Abstract

Alpacas (*Vicugna pacos*) are South American camelids adapted to the high-altitude Andean ecosystem of Peru, above 4,000 m. In this study, semen quality, bacteriological profiles of the prepuce, vagina, and semen collected by postcopulation (PC), artificial vagina (AV), and electroejaculation (EE) methods, and fertility were evaluated in Huacaya alpacas inseminated with fresh PC semen at different times after ovulation induction. A total of 150 Huacaya alpacas (45 males and 105 females) between 4 and 8 years of age were used. Macroscopic and microscopic semen quality parameters, semen bacteriological profiles, and quantitative determination of bacterial load (colony-forming units [CFU]/μL) were evaluated. For these 3 study variables, 30 males were used; these animals were distributed in 3 methods (10 for each method): PC, AV, and EE. In addition, 10 females were included in the postcopulatory method. A total of 10 males and 10 females were used for the bacteriological profile (prepuce and vaginal, respectively). For the fertility test, 80 females (20 for each insemination time) and 5 males (semen donors) were used. Moreover, five females were used for postcopulatory semen collection. Donor semen obtained by the PC method showed significantly higher values for motility (69.1 ± 2.3%), viability (82.8 ± 1.3%), HOST positivity (77.9 ± 1.3%), acrosomal integrity (85.2 ± 1.3%), and seminal volume (2.9 ± 1.8 mL) than those obtained by the AV and EE methods. In contrast, higher viscosity was recorded for AV semen, followed by EE and PC. Regardless of the collection method, the bacteria isolated from the prepuce, vagina, and semen were *Streptococcus* sp., *Staphylococcus* sp., *Escherichia coli*, *Nocardia* sp., *Bacillus* sp., and *Micrococcus* sp. Fertility rates of 45, 50, 35, and 30% were obtained in alpacas inseminated at 24, 26, 28, and 30 h postinduction, respectively, with significant differences between 26 h and the later time points. These results demonstrate significant differences in semen quality parameters and bacterial profiles among collection methods and indicate that 26 h postovulatory induction is the optimal time for artificial insemination with fresh PC semen.

## 1 Introduction

The implementation of various reproductive biotechnologies in South American camelids has been incorporated into different genetic improvement programs in the country, although with poor results (1). Artificial insemination has faced significant technical limitations, mainly related to semen collection, dilution, and cryopreservation, which have restricted its widespread use in genetic improvement programs despite successful birth with both fresh and frozen semen (2). Similarly, marked variability was observed in various seminal parameters in alpacas. In semen collected by artificial vagina, the spermatozoa concentration, Bravo et al. (3) reported values of 56.2 × 10^6^ sperm/mL while Flores et al. (4) reported 17.6 × 10^6^ sperm/mL; in postcopulatory semen, Bravo and Alarcón (5) reported values of 69.0 × 10^6^ sperm/mL; and in vitality, Bravo et al. (3) reported values of 75.2%; Bravo and Alarcón (5) reported 70.8%.

Assessing semen quality, bacteriological profiles, and fertility in reproductive animal studies is critical for ensuring the genetic health and economic efficiency of breeding programs. These parameters allow scientists and breeders to identify reproductive issues, optimize artificial insemination (AI), and prevent the spread of infectious diseases across livestock populations (6–9). In animals, the bacteriological profile of semen strongly influences its quality and reproductive success (fertility). Pathogenic bacteria cause oxidative stress and inflammation, leading to morphological defects, reduced motility, and impaired fertility, whereas beneficial bacteria help maintain optimal sperm parameters (semen quality) (10). Semen bacterial contamination may impair sperm motility, viability, and acrosomal integrity through the production of toxins and reactive oxygen species, potentially reducing fertilization capacity. Therefore, the simultaneous evaluation of bacteriological profiles and fertility outcomes is essential for understanding the relationship between microbial contamination and reproductive efficiency in alpacas. Currently, no studies have simultaneously characterized semen quality, bacteriological profiles across the three collection methods, and fertility outcomes in Huacaya alpacas, representing a relevant knowledge gap in South American camelid reproductive research. Therefore, there is an urgent need to investigate these parameters together in order to improve reproduction in alpacas.

Regarding the bacteriological component, studies in apparently healthy alpacas have identified bacteria of the genera *Escherichia, Bacillus, Staphylococcus, and Streptococcus* in both vaginal samples and preputial swabs (11, 12). In llamas, a similar microbiota has been identified in the uterus, vagina, and both empty and pregnant females and at the preputial level (13). Pezo et al. (14) reported populations of bacteria of *Staphylococcus* spp., *Proteus* spp., *Escherichia coli*, *Streptococcus* spp., *Enterococcus* spp., *Klebsiella* spp., *Gardnerella* spp., *Enterobacter* spp., *Morganella* spp., and *Serratia* spp. in semen collected by artificial vagina and electroejaculation. In another study, Jarvinen and Kinyon (15) identified 22 bacterial genera at the preputial level in alpacas and llamas, with the most frequent being *Actinomyces, Arcanobacterium, Bacillus, Bacteroides, Corynebacterium, Staphylococcus,* and *Streptococcus*. There is no clear knowledge regarding semen bacteriological profiles in alpacas using collection methods (PC, AV, and EE). Understanding the microbiome and bacteriological profile of alpaca semen helps determine the exact causes of idiopathic infertility.

For several decades, reproductive biotechnologies, such as artificial insemination and embryo transfer, have been applied to South American camelids; however, the results have been inefficient. In llamas, various studies on the artificial insemination of semen collected using the artificial vaginal method have reported variable fertility rates. Aller et al. (16) reported 20% fertility, Giuliano et al. (17) achieved 75% fertility, and in alpacas, similarly, the results have been heterogeneous: Gonzáles et al. (18) reported 27.7% fertility with chilled semen, and Ordoñez et al. (19) reported 33.33% fertility with fresh semen from electroejaculation, while García et al. (20) reported 48.4% fertility with fresh semen from postcopulatory ejaculation; Murillo (21) reported 53.33% pregnancy, and García and Alarcón (22) reported 50.5% fertility; Bravo et al. (23) reported that in alpacas and llamas, fertility rates with fresh and thawed semen ranged from 3 to 67%. The optimal time for artificial insemination is not well established for the postcopulation method in alpacas.

Therefore, this study aimed to analyze semen quality, bacteriological profile, and fertility using three semen collection methods, and determine the optimal insemination time using fresh postcopulation semen in Huacaya alpacas (*Vicugna pacos*).

## 2 Materials and methods

### 2.1 Ethical considerations and study location

This study was conducted in accordance with the current legal framework established by Law No. 30407 on animal protection and welfare, which recognizes animals as sentient beings and prohibits mistreatment and abandonment. This study was approved by the Institutional Ethics Committee of the Universidad Nacional Micaela Bastidas de Apurímac under Resolution No. 108-2023-CU-UNAMBA. This research was conducted at the La Raya Experimental Center of the National University of San Antonio Abad of Cusco, located in the district of Marangani, province of Canchis, Cusco.

### 2.2 Semen evaluation

Thirty male alpacas were used and distributed in the 3 collection methods (PC, AV, and EE), 10 for each method. Additionally, 10 females were added to the postcopulatory method. Evaluations of the semen quality parameters (macroscopic and microscopic) obtained through three collection methods, postcopulatory, artificial vagina, and electroejaculation, were performed, and the following variables were analyzed:

*Volume*: Determined by direct visual reading in sterilized collection tubes, expressed in milliliters (mL).

*Color*: Evaluated simultaneously with volume by direct visual observation against light.

*Sperm concentration, motility, and viability*: Analyzed using the CASA Sperm Class Analyzer® (SCA®, Microptic S. L., Barcelona, Spain) computerized semen analysis system.

*Plasma membrane integrity*: Evaluated using the positive Hypo-Osmotic Swelling Test (HOST). A total of 25 μL of the semen sample was incubated in 500 μL of low hypo-osmotic solution (100–150 mOsm/L) for 60 min at 37 °C, and then the sample was analyzed by phase contrast microscopy at 40X, and 200 spermatozoa were counted in several fields of the microscope, where the intact or damaged membrane was evaluated.

*Acrosomal integrity*: Evaluated using Hancock staining.

### 2.3 Bacteriological profile

Ten males and ten females were used for the bacteriological profile (prepuce and vaginal, respectively). Samples for the bacteriological study were obtained by direct swabbing of the prepuce and vaginal walls with sterile swabs, as well as by immersing the swab directly in semen collected using the three methods mentioned. The samples were transported in Cary-Blair medium (Difco®) within polystyrene foam containers on ice, by land and air, to the Veterinary Microbiology and Parasitology Laboratory of the School of Veterinary Medicine at the National University of San Marcos. In the laboratory, the submitted samples were cultured on blood agar and MacConkey agar under both aerobic and anaerobic conditions using Anaerocult anaerobic generators, with an incubation period of 24–72 h at 37 °C. Bacterial identification at the genus and species levels was performed using conventional biochemical tests with support from Bergey’s Manual. The colonies obtained were re-cultured in differential biochemical media, and for bacterial counting, serial dilutions from 10^−1^ to 10^−5^ were carried out using Plate Count Agar (PCA) for mesophilic bacteria and Violet Red Bile Lactose (VRB) agar for coliforms, inoculating 1 mL of each dilution.

### 2.4 Determination of fertility

Semen was collected using the postcopulatory method. Adult female alpacas with live offspring, at least 20 days postpartum, clinically healthy, and receptive to males, were selected. Following collection, the reproductive parameters of the semen were evaluated, and the appropriate dilution was calculated, as well as the presence of preovulatory follicles with diameters ≥ 7 mm. Females deemed suitable for insemination were administered 1 mL of a GnRH analog (0.0042 mg of buserelin acetate) to induce ovulation, recording the exact time of application.

Intrauterine insemination was performed by depositing 1 mL of diluted fresh semen at an approximate concentration of 20 × 10^6^ viable sperm/mL. A total of 80 Huacaya alpaca females (*n* = 20 per group) were divided into four experimental groups as follows:

- G1: 20 female alpacas inseminated 24-h post-ovulation induction.
- G2: 20 female alpacas inseminated 26-h post-ovulation induction.
- G3: 20 female alpacas inseminated 28-h post-ovulation induction.
- G4: 20 female alpacas inseminated 30-h post-ovulation induction.

Fertility was diagnosed 30 days after insemination using transrectal ultrasound to evaluate the presence of a corpus luteum and an implanted embryo.

### 2.5 Analyzed variables

- Semen collection methods: postcopulation, artificial vagina, and electroejaculation.
- Reproductive parameters of semen.
- Bacterial profiles of the vagina, foreskin, and semen.
- Fertility rate.

### 2.6 Statistical analysis

Data on sperm volume, color, concentration, and morphology were analyzed using descriptive statistics, employing measures of central tendency and dispersion. Motility, viability, HOST+ status, and acrosomal integrity percentages were compared using one-way analysis of variance, followed by Duncan’s multiple range test to identify significant differences (*p* ≤ 0.05). Fertility rates were assessed using the non-parametric chi-square (χ^2^) test.

## 3 Results

### 3.1 Semen quality

Macroscopic and microscopic parameters of fresh semen collected using postcopulatory, artificial vagina, and electroejaculation methods were analyzed. The results, shown in Table 1, demonstrate significant differences between the collection methods for the analyzed variables. These variations can be attributed to the specific characteristics of each technique during the semen collection process, which directly influence macroscopic and microscopic seminal parameters.

**Table 1:** Macroscopic and microscopic parameters of semen collected by the postcopulatory, artificial vagina, and electroejaculation methods.

Parameters	Semen collection method		
	Postcopulatory	Artificial vagina	Electroejaculation
Motility (%)	69.1 ^a^ ± 2.3	62.5 ^b^ ± 10.4	59.1 ^c^ ± 1.0
Viability (%)	82.8 ^a^ ± 1.3	71.4 ^b^ ± 3.5	62.0 ^c^ ± 1.2
HOST+ (%)	77.9 ^a^ ± 1.3	67.0 ^b^ ± 1.7	59.7 ^c^ ± 1.2
Acrosomal integrity (%)	85.2 ^a^ ± 1.3	79.8 ^b^ ± 3.0	84.2 ^a^ ± 2.3
Concentration (×10^6^ sperm/mL)	78.5 ^b^ ± 15.1	89.9 ^a^ ± 17.8	62.5 ^b^ ± 20.5
Volume (mL)	2.9 ± 1.8	1.5 ± 0.9	1.6 ± 0.6
Consistency			
Viscosity (%)	10	85	30
Semi-viscosity (%)	90	15	70
Color			
Milky white (%)	13.6	64.6	22.2
Translucent white (%)	4.6	35.4	77.8
Light red (%)	65.9	-	-
Dark red (%)	15.9	-	-

^a–c^Different superscripts within rows indicate significant differences.

### 3.2 Bacteriological profile of the vagina, foreskin, and semen

Seven bacteria were isolated from the foreskin and seven from the vagina. Among these, *Escherichia coli, Enterobacter* sp.*, Staphylococcus* sp.*, S*treptococcus sp., and *Corynebacterium* sp. were isolated. In addition to the five bacteria identified in the foreskin, two additional bacteria were isolated: *Klebsiella oxytoca* and *Citrobacter* sp. In the vagina, two additional bacteria were isolated: *Bacillus* sp. and Providencia sp. These bacteria are native to the environment and may contaminate samples during the sampling process (Table 2).

**Table 2:** Bacteriological profile of the prepuce and vagina of alpacas.

Bacteria	Prepuce	Vagina
*Escherichia coli*	++	+++
*Enterobacter* sp.	++	++
*Staphylococcus* sp.	++	+
*Corynebacterium* sp.	++	+
*Streptococcus* sp.	+	+
*Klebsiella oxytoca*	+	−
*Citrobacter* sp.	+	−
*Bacillus* sp.	−	+
*Providencia* sp.	−	+

+++, High grade; ++, Moderate degree; +, Mild degree; −, Not isolated.

Regarding the bacteria isolated from alpaca semen, despite being considered a clean technique, semen collected by electroejaculation contained five bacteria with a load similar to that of the other methods. Semen obtained from the artificial vagina is usually contaminated with bacteria present on the dummy itself because it is in contact with the ground. Postcopulatory semen, although exposed to external contaminants during copulation, did not show the highest bacterial load, suggesting that factors such as collection techniques and hygiene influence semen contamination (Table 3).

**Table 3:** Bacteriological profile of alpaca semen collected by electroejaculation, post-copulatory, and artificial vagina methods.

Bacteria	Electroejaculation	Postcopulatory	Artificial vagina
*Staphylococcus* sp.	++	+	+
*Streptococcus* sp.	+	++	+
*Escherichia coli*	+	++	−
*Bacillus* sp.	+	+	+
*Nocardia* sp.	+	−	−
*Micrococcus* sp.	−	−	+

+++, High grade; ++, Moderate degree; +, Mild degree; −, Not isolated.

The presence of bacterial colonies (CFU/μL) was quantified in alpaca semen samples obtained using the electroejaculation, postcopulatory, and artificial vagina methods, and the results are presented in Table 4. The analysis showed a higher load of mesophilic bacteria in semen obtained by the postcopulatory method, followed by semen from the artificial vagina, whereas the lowest amount was recorded in samples collected by electroejaculation. The highest population of coliform bacteria was observed in semen obtained using the artificial vagina, followed by electroejaculation, and in smaller quantities in semen collected using the postcopulatory method.

**Table 4:** Quantitative determination of bacterial load expressed in colony-forming units (CFU/μL) in alpaca semen collected by electroejaculation, postcopulatory, and artificial vagina methods.

Collection method	Mesophilic bacteria		Coliform bacteria	
	(CFU/μL)		(CFU/μL)	
	Average	Range	Average	Range
Electroejaculation	13.7 × 10^4^	2 × 10^3^ – 3 × 10^5^	2.37 × 10^4^	1 × 10^2^–8 × 10^4^
Postcopulatory	2.93 × 10^4^	6 × 10^3^–5 × 10^4^	2.83 × 10^3^	1 × 10^3^ – 6 × 10^3^
Artificial vagina	2.41 × 10^4^	7 × 10^2^–6 × 10^4^	2.59 × 10^4^	4 × 10^2^ – 2 × 10^4^

### 3.3 Fertility rate

The fertility rate was independent of the time of insemination (χ^2^, *p* > 0.05), although a maximum trend of 50% fertility was observed at 26 h, followed by 45% at 24 h, 35% at 28 h, and 30% at 30 h. Significant differences were observed between 26 h and the subsequent times of 28 and 30 h, indicating that the optimal time to perform artificial insemination is 26 h post-ovulation induction (Table 5).

**Table 5:** Fertility rate in Huacaya alpacas artificially inseminated with fresh semen collected by the postcopulatory method at different times post-ovulation induction.

Pregnancy condition	Artificial insemination after ovulation induction (hours)							
	24 h		26 h		28 h		30 h	
	*n*	%	*n*	%	*n*	%	*n*	%
Pregnant	9	45^a^	10	50^a^	7	35^b^	6	30^b^
Empty	11	55^a^	10	50^a^	13	65^b^	14	70^b^
Total	20	100	20	100	20	100	20	100

^a,b^Different superscripts within rows indicate significant differences.

## 4 Discussion

Semen obtained by postcopulation showed the highest percentages of sperm viability, HOST-positive status, and acrosomal integrity, with significant differences compared to the other methods, although there were no differences in motility. This is attributed to the fact that postcopulatory semen is less viscous, semi-dilute, and contains vaginal and uterine fluids that could protect the sperm during their passage to the uterotubal junction, thus improving fertilization (22, 24).

The semen motility obtained using an artificial vagina was lower than that reported by Bravo et al. (3) who reported 75.2% and by Bravo and Alarcón (5), with 69%, while the motility of the semen collected by the postcopulatory method was similar to those described by Bravo and Alarcón and García and Alarcón (5, 22). However, sperm vitality and positive HOST results were higher than those reported by Bravo et al., Bravo and Alarcón, and García and Alarcón (3, 5, 22).

Sperm concentration showed significant differences, with the highest concentration in semen obtained by artificial vagina, followed by postcopulation and electroejaculation. This higher concentration of semen from the artificial vagina is because the semen is the entire ejaculate, whereas the semen from electroejaculation is variable because it depends largely on the disposition of the male, and the semen from postcopulation is diluted with vaginal and uterine fluids and is incomplete, as only a fraction of the ejaculate is recovered at the vaginal level (20, 25). The sperm concentration values obtained from semen collected by the postcopulatory method (78.5 × 10^6^ sperm/mL) coincide with those reported by Bravo and Alarcón (5) (75.20 × 10^6^ sperm/mL) and García and Alarcón (22) (73.8 × 10^6^ sperm/mL). In contrast, the concentration of semen collected by artificial vagina (89.9 × 10^6^ sperm/mL) exceeded that reported in alpacas: 17.6 × 10^6^ sperm/mL (4), 56.2 × 10^6^ sperm/mL (3), and 80.34 × 10^6^ sperm/mL (5).

Seminal volume was higher in semen obtained postcopulatorily; this result is attributed to the additional presence of blood and vaginal and uterine fluids. García and Alarcón (22) reported similar values in semen obtained by the postcopulatory method of 2.8 mL, while Bravo and Alarcón (5) reported values higher than 3.6 mL because the seminal volume obtained is the average of that collected from alpacas and llamas, respectively. Meanwhile, volumes between 1.4 and 1.8 mL are reported for semen collected using an artificial vagina (3–5, 26). These ranges coincide with the values obtained in the current investigation. This variability in semen volume could be due to the frequency of use of males in natural mating, as well as the frequency of semen collection.

Regarding semen viscosity, the semen collected using the artificial vagina method showed a higher viscosity, attributed to the presence of fluids rich in mucopolysaccharides from the bulbourethral glands and prostate, which gives the semen of South American camelids its characteristically high viscosity. While the semen obtained by electroejaculation showed a highly variable viscosity from one male to another, the semen collected by the postcopulatory method had a lower viscosity. The results obtained in the present study agree with those reported in other studies carried out on alpacas, which indicate that semen collected using an artificial vagina has high viscosity, a characteristic of this species (3–5, 26). Similarly, other studies in alpacas, with semen obtained by the postcopulatory method, reported that they are also slightly viscous, similar to the results obtained (5, 27).

Semen collected using the postcopulatory method showed a variation in coloration, from light red to dark red, attributed to the presence of erythrocytes from the blood that are incorporated into the semen by the constant friction of the penis against the endometrial walls of the vagina and uterus during intrauterine copulation. The semen obtained by the artificial vagina method was mostly milky white, and the semen collected by the electroejaculation method showed a predominantly translucent white color, which was due to the lower amount of fluids from the accessory glands. Reports of semen color collected using the artificial vagina method range from translucent white to milky white (3–5, 26), similar to the results obtained. Moreover, studies have reported that semen collected by the postcopulatory method has reddish coloration (5) and light red coloration (22).

From the study of the bacteriological profile of the foreskin and vagina, several bacteria were isolated that can be considered common resident bacteria in these two organs. In alpacas, Pacheco et al. (11) reported similar bacteria identified at the preputial level, although with a higher bacterial load; they also noted the presence of the fungus *Aspergillus fumigatus*. In boar semen, Ciornei et al. (28) identified 9 fungal genera, with *Candida parapsilosis* being the most prevalent (92.1%), followed by Geotrichum candidum (72.3%), Aspergillus spp., and Penicillium spp. (63.3%); filamentous fungi originate from ambient air, while yeasts are of endogenous origin (28). The current study did not include a mycological evaluation of alpaca semen, which constitutes a limitation and a priority area for future research. Ciornei et al. (29) confirmed that 37.4% of 24 microbial genera identified in fresh semen were fungi (9 out of 15 genera), with a fungus-to-bacteria ratio of 1:1.6. *Candida parapsilosis* and *C. sake* accounted for 92.1% of fresh semen and 94.1% of semen diluted at 24 h, whereas most bacteria decreased to negligible levels. Given that Pacheco et al. (11) have already reported the presence of the fungus *Aspergillus fumigatus* in alpaca semen, it is likely that contamination by yeasts such as Candida spp. is also present and persists during storage, representing a high risk of AI doses for alpacas (29). At the vaginal level, of the seven bacteria isolated, six coincided with those of the present study, and only *Streptococcus* sp. was not reported. In llamas, Jimenez (13) reported the presence of thirteen bacteria at the vaginal level, of which six bacteria are the same as those identified in this investigation; likewise, three of the ten bacteria described by Jarvinen and Kinyon (15) coincide with the results obtained. However, these authors reported a greater diversity of bacteria than found in the present study; consequently, the vaginal bacterial microbiota resident in alpacas is also present in llamas.

In alpaca semen collected using electroejaculation, artificial vagina, and postcopulatory methods, Pacheco et al. (11) reported the presence of bacteria similar to those in the current study but with a high bacterial load. In alpaca semen obtained using an artificial vagina, *Bacillus* sp., *Micrococcus* sp., *Staphylococcus* sp., and *Streptococcus* sp. were isolated, and in llamas, in addition to these four bacteria, *Enterobacter* sp., *Enterococcus* sp., *Gardnerella* sp., *Klebsiella* sp., *Morganella* sp., and *Proteus* sp. were isolated (14). Similarly, bacterial load has been reported in semen collected by the artificial vagina method in sheep (30, 31), as well as in cattle (32).

In a study using llamas, *Bacillus* sp., *Escherichia coli*, *Staphylococcus* sp., and *Streptococcus* sp. were isolated from semen collected via electroejaculation. In another study using semen obtained with an artificial vagina, in addition to these bacteria, they found *Enterobacter* sp., *Enterococcus* sp., *Gardnerella* sp., *Klebsiella* sp., *Morganella* sp., *Proteus* sp., and *Serratia* sp. (14). The high number of bacteria reported could be related to the animal species and location where these studies were conducted.

In semen obtained using the postcopulatory method, where the bacteria present in the collected sample come from both males and females, *Bacillus* sp., *Escherichia coli*, *Staphylococcus* sp., and *Streptococcus* sp. were isolated. These are common resident bacteria found in the reproductive tracts of male and female sheep and cattle (33), horses (34), and camelids (11, 13, 14, 35). A high bacterial load compromises sperm viability and, therefore, semen quality (31).

The mesophilic bacterial load was highest in semen samples obtained by electroejaculation, followed by semen collected postcopulatory and via the artificial vagina, likely due to a greater degree of manipulation of the prepuce and penis before and during semen collection; meanwhile, the coliform bacterial load showed no significant differences among the three semen collection methods. Previous studies in alpacas by Pacheco et al. (11) and in sheep (31) reported higher bacterial loads than those found in the present study; conversely, a lower bacterial load has been described in boar semen (36).

Ciornei et al. (37) identified in fresh boar semen the same bacterial genera recovered in the present study from alpaca semen: *Escherichia coli*, *Staphylococcus* spp., *Streptococcus* spp., and *Bacillus* sp., suggesting that primary contamination sources (preputial flora, skin, collection equipment, and ambient air) are the same across phylogenetically distant livestock species and that standardized microbiological control measures are essential in any assisted reproductive technology program. Additionally, while antibiotic-supplemented extenders effectively reduce the bacterial load, they lack antifungal activity, allowing *Candida parapsilosis* to persist and proliferate during storage by utilizing glucose as an extender. Since alpaca diluents contain similar sugars, future studies should incorporate mycological profiling of alpaca semen to assess this potential risk at AI doses (37).

From an applied perspective, Althouse (38) indicated that while the use of antibiotics in semen diluents is the standard practice for controlling bacterial contamination, emerging antimicrobial resistance poses a growing risk: 96.1% of bacterial isolates in diluted semen belonged to a single species with intrinsic resistance to the antibiotic in the diluent (38). As an alternative, this author identified the use of antimicrobial peptides, metal nanoparticles, natural compounds, and storage at reduced temperatures as promising strategies for reducing the microbial load without depending exclusively on antibiotics. This review reinforces the importance of implementing routine microbiological monitoring protocols and risk-mitigation strategies in any AI program, including those developed for alpacas (38).

Ciornei et al. (28) demonstrated that standardized hygiene (HPBC) effectively reduced bacterial contamination in boar semen, but not fungal contamination, and that adding 12.5% fluconazole to diluents achieved complete sterilization without compromising fertility. These results reinforce the need to evaluate the mycological profile of alpaca semen and incorporate antifungal strategies into semen processing protocols for this species (28).

Althouse (39) emphasized that routine microbiological monitoring (at least 20% of batches per day) and verification of purified water quality are critical for ensuring the biosafety of AI doses (39). Furthermore, he documented cases in which impurities in commercial diluents significantly reduced sperm viability and farrowing rates on swine farms, underscoring that input suppliers must have robust quality control and quality assurance (QC/QA) programs that include sperm safety testing. For alpacas, where semen is processed and diluted under conditions that are often handcrafted and lack standardized input control protocols, these findings justify the implementation of formal quality monitoring systems that minimize the risk of biological and chemical contamination in artificial insemination doses (39).

Regarding the effect of mycospermia on fertility, Ciornei et al. (29) inseminated sows with known doses of mycospermia: the group with the lowest fungal load (0.140 × 10^3^ LogCFU/mL; 12 h) achieved 86% fertility and 9.63 piglets per litter, compared to 82% and 9.56 in the group with the higher load (0.236 × 10^3^ LogCFU/mL; 24 h). Although the difference was not statistically significant (*p* = 0.279), the negative trend was relevant; higher Candida spp. load and longer storage time were associated with lower reproductive rates. This finding is particularly relevant for alpacas, where artificial insemination is performed with fresh semen stored for a short time, and the diluents contain sugars that could promote yeast growth. The lack of studies on mycospermia in alpacas represents a knowledge gap for future research to address, analyzing both the frequency of isolation and the potential effect of yeasts on sperm viability and fertility in this species (29).

Studies on artificial insemination of alpacas using frozen semen obtained from the vas deferens reported low fertility rates of 0, 15.78, and 25% when females were inseminated 24, 26, and 30 h post-ovulation induction, respectively. These lower fertility rates were observed compared to those in the current study, which may be attributed to the use of frozen semen, given that freezing techniques are not yet fully standardized in this species. Additionally, only sperm and diluent were used, without the presence of accessory gland fluids, which could have affected the fertilization capacity. The highest fertility rate was obtained 30 h after post-ovulation induction (40). In another study of insemination in alpacas, using frozen semen from an artificial vagina, pregnancy rates were lower than those obtained in the present study: 16.6, 38.8, and 27.7%, when inseminated at 26, 28, and 30 h post-ovulation induction, respectively. The higher pregnancy rate was obtained 28 h after ovulation induction (18).

In previous studies on artificial insemination in alpacas, using fresh semen collected by the postcopulatory method, Murillo (21) reported a fertility rate of 53.33% when insemination was performed 28 h post-ovulation induction. This value exceeds that obtained in the present study for the same time but is similar to the rate obtained at 26 h post-ovulatory induction. In alpacas inseminated within a range from 26 to 30 h after ovulatory induction, García et al. and García and Alarcón (20, 22) reported fertility rates of 50 and 50.5%, respectively; those results are similar to those found in the present study when insemination was performed 26 h post-ovulation induction.

## 5 Conclusion

Semen collection methods using postcopulation, artificial vagina, and electroejaculation significantly influence semen quality parameters, affecting variables such as motility, sperm viability, HOST+ status, acrosomal integrity, sperm concentration, semen volume, viscosity, and color.

Mesophilic and coliform bacteria were identified in the foreskin, vagina, and semen, with variable profiles and loads depending on the collection method; these bacteria are considered part of the resident microbiota.

The optimal time for artificial insemination with fresh semen collected by the postcopulation method to achieve fertility rates above 50% is 26 h after post-ovulation induction.

## Data Availability

The raw data supporting the conclusions of this article will be made available by the authors, without undue reservation.

## References

[1] Huanca W. Biotecnologías reproductivas en camélidos sudamericanos domésticos como alternativas para la mejora genética. Arch Latinoam Prod Anim. (2015) 23:4

[2] Adams GP Ratto MH Collins CW Bergfelt DR. Artificial insemination in south American camelids and wild equids. Theriogenology. (2009) 71:166–75. doi: 10.1016/j.theriogenology.2008.09.005, 18922569

[3] Bravo W Flores D Ordoñez C. Effect of repeated collection on semen characteristics of alpacas. Biol Reprod. (1997) 57:520–4. doi: 10.1095/biolreprod57.3.520, 9282985

[4] Flores P García-Huidobro J Muñoz C Bustos-Obregón E Urquieta B. Alpaca semen characteristics previous to a mating period. Anim Reprod Sci. (2002) 72:259–66. doi: 10.1016/S0378-4320(02)00095-7, 12137987

[5] Bravo W Alarcon V. Preservación de semen y avances recientes en la inseminación artificial de llamas y alpacas Semen preservation and recent advances in artificial insemination of llamas and alpacas. SPERNOVA. (2013) 3:3

[6] Said S Diansyah AM Rahayu JD Maulana T Gunawan M Kaiin EM. A comparative analysis of semen quality traits and sperm kinematic parameters in relation to fertility prediction in Murrah buffaloes. J Adv Vet Animal Res. (2025) 12:751. doi: 10.5455/javar.2025.l938, 41328239 PMC12665311

[7] Tanga BM Qamar AY Raza S Bang S Fang X Yoon K . Semen evaluation: methodological advancements in sperm quality-specific fertility assessment — a review. Anim Biosci. (2021) 34:1253–70. doi: 10.5713/ab.21.0072, 33902175 PMC8255896

[8] Lenický M Slanina T Kačániová M Galovičová L Petrovičová M Ďuračka M . Identification of bacterial profiles and their interactions with selected quality, oxidative, and immunological parameters of Turkey semen. Animals. (2021) 11:1771. doi: 10.3390/ani11061771, 34198509 PMC8231993

[9] Khan MZ Chen W Naz S Liu X Liang H Chen Y . Determinant genetic markers of semen quality in livestock. Front Endocrinol. (2024) 15:1456305. doi: 10.3389/fendo.2024.1456305, 39429738 PMC11489916

[10] Sorkytė Š Šiugždinienė R Virgailis M Vaičiulienė G Wysokińska A Wójcik E . The interaction between canine semen bacteria and semen quality parameters. Animals. (2024) 14:2151. doi: 10.3390/ani14152151, 39123677 PMC11311067

[11] Pacheco J Angulo J Siuce J García W. Determinación de bacterias y hongos en semen fresco de alpaca obtenido por tres técnicas de colección. Rev Investig Vet Peru. (2022) 33:e22911. doi: 10.15381/rivep.v33i3.22911

[12] Tibary A Anouassi a. Uterine infections in camelidae. Vet Sci Tomorrow. (2001) 3:12.

[13] Jimenez J. (2016) Detrerminacion de la flora bacteriana de la vagina y utero, y la realcion con la fertilidad en camelidos sudamericanos domesticos (*Lama glama*) del Centro Experimental Agropecuario Condoriri La Paz - Bolivia Universidad Mayor de San Andres. Available online at: http://repositorio.umsa.bo/xmlui/handle/123456789/13285

[14] Pezo D. Meza A. Ampuero E. Pezo S. Cardenas N. Becerra J. Evaluación bacteriológica de semen de llamas reproductores obtenidos mediante vagina artificial y electroeyaculación. VI Jornadas Internacionales Instituto de Investigación y Tecnología en Reproducción Animal INITRA. (2021). 87

[15] Jarvinen J Kinyon J. Preputial microflora of llamas (*Lama glama*) and alpacas (*Vicugna pacos*). Small Rumin Res. (2010) 90:156–60. doi: 10.1016/j.smallrumres.2010.01.007, 38826717

[16] Aller J Ferré L Rebuffi G Alberio R. Inseminación artificial en llamas (*Lama glama*). Primera comunicación en Argentina. Jujuy, Argentina: Sitio Argentino de Producción Animal (1997). p. 5.

[17] Giuliano S Chaves M Trasorras V Gambarotta M Neild D Director A . Development of an artificial insemination protocol in llamas using cooled semen. Anim Reprod Sci. (2012) 131:204–10. doi: 10.1016/j.anireprosci.2012.03.010, 22503638

[18] Gonzáles M. Huanca T. Cárdenas O. (2011) Evaluación de la fertilidad en alpacas inseminadas con semen refrigerado a diferentes tiempos post inducción de ovulación. Spermova. Asociación Peruana de Reproducción Animal; 102–103. Available online at: https://repositorio.inia.gob.pe/handle/20.500.12955/1591

[19] Ordoñez C Cucho H Ampuero E Antezana Julián W Cayo S. Inseminación artificial de alpacas con semen fresco, refrigerado y descongelado colectado por electroeyaculación artificial insemination with fresh, cooled and thawed alpaca semen collected with electroejaculation. SPERMOVA Spermova. (2013) 3:65–6.

[20] García W Alarcón V Bravo W. Inseminación Artificial de Alpacas con Semen Refrigerado y con Inclusión de Dos Tipos de Yema de Huevo. Rev Investig Vet Peru. (2017) 28:337. doi: 10.15381/rivep.v28i2.13080

[21] Murillo Y. Tasa de fertilidad a la inseminación artificial y mérito económico en alpacas huacaya. Revista de Investigaciones. (2018) 7:4.

[22] García W Alarcón V. Efecto de la inseminación artificial con semen fresco, con uno y dos servicios, en la fertilidad y natalidad de las alpacas. Rev Investig Vet Peru. (2019) 30:760–7. doi: 10.15381/rivep.v30i2.16072

[23] Bravo PW Alarcon V Baca L Cuba Y Ordoñez C Salinas J . Semen preservation and artificial insemination in domesticated south American camelids. Anim Reprod Sci. (2013) 136:157–63. doi: 10.1016/j.anireprosci.2012.10.005, 23153624

[24] Hafez E. Hafez B. (2002) Casadellibro. Reproducción e inseminación artificial en animales (7a ED.) | VV.AA. | Casa del Libro. Available online at: https://www.casadellibro.com/libro-reproduccion-e-inseminacion-artificial-en-animales-7-ed/9789701037195/825228

[25] Alarcón V García W Bravo W. Inseminación artificial de alpacas con semen colectado por aspiración vaginal y vagina artificial. Rev Investig Vet Peru. (2012) 23:58–64. doi: 10.15381/rivep.v23i1.882

[26] Garnica J Achata R Bravo W. Physical and biochemical characteristics of alpaca semen. Anim Reprod Sci. (1993) 32:85–90. doi: 10.1016/0378-4320(93)90059-Z

[27] Sumar J Garcia M. Nuclear and related techniques in animal production and health. Livest Prod Sci. (1986) 17:382. doi: 10.1016/0301-6226(87)90088-1

[28] Ciornei Ş Drugociu D Ciornei LM Mareş M Roşca P. Total aseptization of boar semen, to increase the biosecurity of reproduction in swine. Molecules. (2021) 26:6183. doi: 10.3390/molecules26206183, 34684764 PMC8541232

[29] Ciornei ȘG Drugociu D Roşca P. *Candida* genus maximum incidence in boar semen even after preservation, is it not a risk for AI though? Molecules. (2022) 27:7539. doi: 10.3390/molecules27217539, 36364363 PMC9656137

[30] Ahmed E Islam M Alam M Jha P Ghosh S Naher N . Bacterial contamination of ram semen used for artificial insemination in indigenous ewes. Bangl Vet. (2017) 34:20–6. doi: 10.3329/bvet.v34i1.38709

[31] Yániz J Marco-Aguado MA Mateos J Santolaria P. Bacterial contamination of ram semen, antibiotic sensitivities, and effects on sperm quality during storage at 15°C. Anim Reprod Sci. (2010) 122:1–9. doi: 10.1016/j.anireprosci.2010.08.006, 20832206

[32] Meena G Raina V Gupta A Mohanty T Bhakat M Abdullah M . Effect of preputial washing on bacterial load and preservability of semen in Murrah buffalo bulls. Vet World. (2015) 8:798–803. doi: 10.14202/vetworld.2015.798-803, 27065650 PMC4825285

[33] Swartz J Lachman M Westveer K ONeill T Geary T Kott R . Characterization of the vaginal microbiota of ewes and cows reveals a unique microbiota with low levels of lactobacilli and near-neutral pH. Front Vet Sci. (2014) 1:19. doi: 10.3389/fvets.2014.00019, 26664918 PMC4672155

[34] Clément F Vidament M Guérin B. Microbial contamination of stallion semen. Biol Reprod. (1995) 52:779–86. doi: 10.1093/biolreprod/52.monograph_series1.779

[35] Mshelia G Okpaje G Voltaire Y Egwu G. Comparative studies on genital infections and antimicrobial susceptibility patterns of isolates from camels (*Camelus dromedarius*) and cows (*Bos indicus*) in Maiduguri, North-Eastern Nigeria. Springerplus. (2014) 3:91. doi: 10.1186/2193-1801-3-91, 24570857 PMC3933609

[36] Bennemann P Machado S Girardini L Sonálio K Tonin A. Contaminantes bacterianos y perfil de susceptibilidad del semen porcino en centros de recogida em Brasil. Rev MVZ Cordoba. (2018) 23:6637–48. doi: 10.21897/rmvz.1338

[37] Gregore CS Roşca P Drugociu D. Bacterial and fungal burden in boar semen. Res J Biotechnol. (2012) 7:23–7.

[38] Althouse GC. Contaminant toxicity of concern for boars and semen used in assisted reproduction programs. Anim Reprod Sci. (2024) 269:107519. doi: 10.1016/j.anireprosci.2024.107519 38897823

[39] Althouse G. Biological and chemical contaminants in extended porcine semen: outcomes and diagnosis. Anim Reprod Sci. (2022) 247:107086. doi: 10.1016/j.anireprosci.2022.107086, 36191426

[40] Pacheco J Perez G Calle L Garcia W. Efecto del lugar y la hora de inseminacion artificial sobre la fertilidad en alpacas (effect of the place and the hour of artificial insemination on the fertility in alpacas). REDVET. (2009) 10:5.

